# Stimulation of Na^+^/K^+^-ATPase with an Antibody against Its 4^th^ Extracellular Region Attenuates Angiotensin II-Induced H9c2 Cardiomyocyte Hypertrophy via an AMPK/SIRT3/PPAR*γ* Signaling Pathway

**DOI:** 10.1155/2019/4616034

**Published:** 2019-09-15

**Authors:** Siping Xiong, Hai-Jian Sun, Lei Cao, Mengyuan Zhu, Tengteng Liu, Zhiyuan Wu, Jin-Song Bian

**Affiliations:** ^1^Department of Pharmacology, Yong Loo Lin School of Medicine, National University of Singapore, Singapore 117597; ^2^National University of Singapore (Suzhou) Research Institute, Suzhou, China

## Abstract

Activation of the renin-angiotensin system (RAS) contributes to the pathogenesis of cardiovascular diseases. Sodium potassium ATPase (NKA) expression and activity are often regulated by angiotensin II (Ang II). This study is aimed at investigating whether DR-Ab, an antibody against 4^th^ extracellular region of NKA, can protect Ang II-induced cardiomyocyte hypertrophy. Our results showed that Ang II treatment significantly reduced NKA activity and membrane expression. Pretreatment with DR-Ab preserved cell size in Ang II-induced cardiomyopathy by stabilizing the plasma membrane expression of NKA and restoring its activity. DR-Ab reduced intracellular ROS generation through inhibition of NADPH oxidase activity and protection of mitochondrial functions in Ang II-treated H9c2 cardiomyocytes. Pharmacological manipulation and Western blotting analysis demonstrated the cardioprotective effects were mediated by the activation of the AMPK/Sirt-3/PPAR*γ* signaling pathway. Taken together, our results suggest that dysfunction of NKA is an important mechanism for Ang II-induced cardiomyopathy and DR-Ab may be a novel and promising therapeutic approach to treat cardiomyocyte hypertrophy.

## 1. Introduction

Cardiovascular disorders are one of the most common diseases in adults and the leading cause of death worldwide [1]. Pathological activation of renin-angiotensin system (RAS) is a keyfactor in several cardiovascular diseases [2]. Angiotensin (Ang) II, a critical component of RAS, presents in both systemic circulation and local organs such as the brain, blood vessel, kidney, and heart [2, 3]. Multiple studies reported that increased Ang II leads to hypertension and also directly promotes cardiomyocyte death, hypertrophy, and remodeling [2]. They have proved that Ang II is involved in cardiomyocyte damage [4–6]. Unscrambling the underline mechanisms of Ang II may supply a new therapeutic target for the prevention and treatment of these diseases.

In most mammalian cells, sodium potassium ATPase (NKA) is an energy-transducing ion pump across the plasma membrane [7]. In the past decade, NKA has also been proved to be an ion-pumping-independent receptor function that confers a ligand-like effect of cardiotonic steroids (CTS) on protein/lipid kinases, intracellular Ca^2+^ oscillation, and ROS production [8, 9]. However, drugs targeted at NKA are mainly CTS which was used to treat chronic heart failure, a kind of cardiovascular diseases. These chemical drugs also often cause severe toxic effects, such as cardiac arrhythmias and atrioventricular block, gastrointestinal disorders, nervous system disorders, anorexia, blurred vision, nausea, and vomiting [10]. In recent years, we and other groups have demonstrated that antibody targeted at DR region (897DVEDSYGQQWTYEQR911, amino acid sequence number showed as in rat), the 4^th^ extracellular domain of *α*-subunit of NKA, can activate NKA's function [10, 11]. Our previous studies have already proved that DR-Ab produces cardioprotection and protects isoproterenol-induced mouse cardiac injury [10, 12]. Therefore, this antibody was a kind of ideal tool to study the NKA function in relative studies.

Recently, extensive studies have demonstrated that Ang II has a close relationship with NKA. Rasmussen's group reported that Ang II induced NKA inhibition in cardiac myocytes via PKC-dependent activation of NADPH oxidase [13]. Massey et al. also reported that Ang II-dependent phosphorylation of the rat kidney NKA at specific sites can regulate how the NKA releases bound cardiac glycoside [14]. Moreover, Ang II inhibits the NKA activity accompanied with the involvement of an increase in NADPH oxidase-derived O_2_^∗^- [15]. Thus, the present study was designed to study the effects of DR-Ab in Ang II-induced cardiac myocyte damage and its underlying mechanism.

## 2. Material and Methods

### 2.1. Chemicals and Reagents

Antibodies against p22^phox^, p47^phox^, Na^+^/K^+^-ATPase alpha 1 (NKA *α*1), PPAR-*γ*, Sirt-3, *β*-actin, GAPDH, *β*-tubulin, and the horseradish peroxidase-conjugated secondary antibodies were purchased from Santa Cruz Biotechnology Inc. (Santa Cruz, CA, USA). Antibodies against phosphorylated and total AMPK were purchased from Cell Signaling Technology (Beverly, MA, USA). The specific primers were synthesized by Integrated DNA Technologies Pte. Ltd. (Singapore). Antibody against *α*-actinin was obtained from Abcam (Cambridge, MA, USA). Mitochondrial membrane potential assay kit with JC-1 and the kits for measurement of ATP were purchased from Beyotime Institute of Biotechnology (Shanghai, China). Dihydroethidium (DHE) and 2′,7′-dichlorofluorescin diacetate (DCFH-DA) were purchased from Sigma-Aldrich (St. Louis, MO, USA). MitoSOX™ was purchased from Invitrogen (Carlsbad, CA, USA). AMPK inhibitor compound C, a selective Sirt3 inhibitor 3-TYP, and PPAR*γ* antagonists GW9662 were obtained from Cayman Chemical Company (Ann Arbor, MI, USA). DR-Ab was generated and identified in our lab as previously described [12, 16].

### 2.2. Cell Culture

Embryonic rat heart-derived cells (H9c2, passage 15) preserved by our lab were cultured in high-glucose Dulbecco's modified Eagle's medium (4.5 g/l glucose) supplemented with 10% fetal bovine serum (FBS) and 1% antibiotics (penicillin-streptomycin, Gibco) in humidified air containing 5% CO_2_ at 37°C.

Primary neonatal mouse cardiomyocytes: the cardiomyocytes were isolated from 1- to 3-day-old C57BL/6 neonatal mice as described previously [17–19]. In short, the hearts were placed into ice-cold Hanks' balanced saline solution (HBSS; Life Technologies). After removal of atrial and aortic appendages, the cardiomyocytes were collected by using 0.2 mg/ml collagenase type II (Worthington Biochemical, Lakewood, NJ) and 0.6 mg/ml pancreatin (Sigma, MAK030, St. Louis, MO) at common cellular incubator. The supernatant-containing suspended cells were cultured in minimum essential medium with 10% fetal bovine serum for 2 h to remove nonmyocytes. Then, the culture medium was changed to minimum essential medium containing 10% FBS with 1% antibiotics after seeding for 48h. Cardiomyocytes were seeded 3 days prior to use.

All primary cell culture protocols were performed strictly according to the principles and guidance of Institutional Animals Care and Use Committee at the National University of Singapore.

### 2.3. Intracellular and Mitochondrial ROS Measurement

After fixing collected H9c2 cells, they were incubated with DHE (10 *μ*M) and DCFH-DA (10 *μ*M) in a dark and humidified incubator at 37°C for 30min as previously described [20] and changed the solution to phosphate-buffered saline (PBS) and observed on microscope immediately.

Mitochondrial ROS production was measured with a fluorogenic dye named MitoSOX Red (Invitrogen, Darmstadt, Germany). Cells were loaded with 1 *μ*M MitoSOX Red for 30 min at 37°C protecting from light and washed cells with PBS and then observed on microscope (DMi 8; Leica, Microsystems, Germany).

The fluorescence signals were captured and analyzed with the Image-Pro Plus 6.0 (Version 6.0, Media Cybernetics, Bethesda, MD, USA) in same parameters.

### 2.4. Western Blotting Analysis

After washing twice with PBS, the cells were lysed with ice-cold lysis buffer. The cell lysate was centrifuged at 10,000 g for 10 min at 4°C. Equal amount of proteins was electrophoresed, transferred, blotted, and then incubated with required primary antibodies at 4°C overnight. After washing with TBST buffer three times, the membranes were incubated with appropriate secondary horseradish peroxidase- (HRP-) conjugated antibodies. Then, membranes were detected using an ECL Advanced Western Blot Detection Kit (Millipore Darmstadt, Germany). The integrated optical density was quantified with the Image-Pro Plus 6.0 software.

### 2.5. Measurement of Mitochondrial Membrane Potential

Mitochondrial membrane potential was dectected with 5,5′,6,6′-tetrachloro-1,1′,3,3′-tetraethylbenzimidazolcarbocyanine iodide (JC-1) (Beyotime Institute of Biotechnology, Shanghai, China). The H9c2 cells were stained with JC-1 and observed with a fluorescence microscope (DMi 8; Leica, Microsystems, Germany).

### 2.6. Real-Time PCR

Total RNA extraction was performed with TRIzol (Life Technologies, USA) according to the manufacturer's instructions, and then RevertAid First-Strand cDNA Synthesis Kit (Thermo Scientific, USA) was used for reverse transcription. Following, GoTaq® quantitative PCR (qPCR) Master Mix (Promega, USA) was used for quantitative PCR with indicated primers on a VIIA(TM) 7 System (Applied Biosystems). Data were analyzed by normalization against GAPDH. The primers used are indicated as in Table 1.

### 2.7. Plasma Membrane Extraction

EZ-Link NHS-SS-biotin (Pierce Chemical Co., USA) was used to label surface protein for 1 h. Cells were washed with PBS containing 100 mM glycine and then lysed in lysis buffer. After protein quantitative, equal proteins (150–300 *μ*g) were added to Streptavidin (Pierce Chemical Co.) beads at 4°C overnight. Next day, beads were washed thoroughly, resuspended in 30 *μ*l loading buffer, and analyzed using Western blots.

### 2.8. Isolation of Endosomes

The preparation of endosomes was fractioned on a floatation gradient. In brief, the treated cells were washed by cold PBS and homogenization buffer (250 mM sucrose and 3 mM imidazole, pH 7.4). After centrifuging for 10 min at 2000×g in 4 °C, the supernatant was adjusted to 40.6% sucrose, followed by incubation of 35% sucrose supplemented with 3 mM imidazole and 0.5 mM EDTA and homogenization buffer. The samples were centrifuged at 210,000×g for 1.5 h; the endosomes were then obtained at the homogenization buffer—35% sucrose interface. The endosome fraction was identified by immunoblots for Rab 7 as previously described [12, 21].

### 2.9. Measurement of NKA Activity

NKA activity was determined according to previous study [22, 23]. H9c2 cells were homogenized in buffer A containing 20 mM HEPES, 250 mM sucrose, 2 mM EDTA, 1 mM MgCl2, pH 7.4, and then centrifuged at 20,000 g for 30 min. Consequently, resuspended the pellet in buffer A again and quantified the protein. One 50 *μ*l aliquot of homogenate was mixed with 50 *μ*l of reaction buffer 1 (200 mM Tris-HCl, 30 mM MgCl2, 200 mM NaCl, 60 mM KCl, 10 mM EGTA, pH 7.5). Another 50 *μ*l aliquot was mixed with reaction buffer 2 (buffer 1+1 mM ouabain). To prevent protein degradation, 100 *μ*g/ml PMSF, 2 *μ*g/ml apronitin, and 2 *μ*g/ml pepstatin A were added in. After 1 mM of ATP was added, the mixtures were incubated for 10 min at 37°C and then stopped by adding 10 *μ*l of 100% (*w*/*v*) trichloroacetic acid. After incubating them on ice for 1 h, they were centrifuged at 20,000 g for 30 min. The supernatant without phosphate was assayed with the Phosphate Colorimetric Kit (Sigma, MAK030, St. Louis, MO) at 650 nm.

### 2.10. Immunofluorescence Staining

Immunofluorescent staining was performed as described previously [24]. The collected H9c2 cardiomyocytes or primary neonatal mouse cardiomyocytes were fixed in freshly made -20°C ethanol at room temperature for 10 min and then permeabilized with 0.1% Triton X-100. After blocking with 5% BSA at room temperature for 1 h, the cells were incubated with the mouse anti-NKA antibody or mouse anti-*α*-actinin overnight at 4°C. Next, the cells were washed with PBS three times, and then incubated with goat anti-mouse cross-adsorbed secondary antibody, Alexa Fluor 488 (Invitrogen, Carlsbad, CA, USA) for 1 h at room temperature, and the nucleus was stained with DAPI. Goat anti-mouse IgG (H+L) cross-adsorbed secondary antibody, Alexa Fluor 488 (Invitrogen, Carlsbad, CA, USA). The images were captured with a fluorescence microscope (Leica DMi8, Leica, Wetzlar, Germany).

### 2.11. Statistical Analysis

Data were expressed as mean ± SD. One-way or two-way ANOVA followed by the post hoc Bonferroni test was used for multiple comparisons. A value of *p* < 0.05 was considered statistically significant.

## 3. Results

### 3.1. DR-Ab Improves Ang II-Induced Cardiomyocyte Hypertrophy through Preservation of NKA Activity

**Figure 1:**
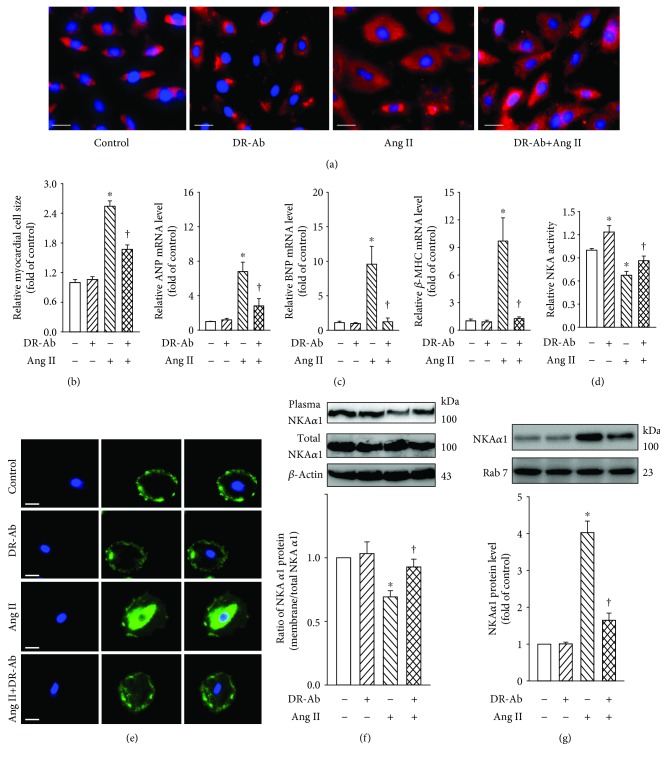
Effects of DR-Ab on Ang II-induced H9c2 cardiomyocyte hypertrophy. DR-Ab (2 *μ*M) was given 30 min before treatment with Ang II (100 nM) for 48 h. (a, b) Representative immunofluorescence staining (a) and group data (b) showing that DR-Ab reversed enlarged cell size caused by Ang II. Red: *α*-actinin. Blue: DAPI. Scale bar, 25 *μ*m. *n* = 6. (c) qRT-PCR analysis showing the mRNA levels of ANP, BNP, and *β*-MHC. *n* = 4. (d–g) DR-Ab reversed Ang II-induced loss of plasma membrane NKA *α*1 (e, f), increase of endosome NKA *α*1 (g), and downregulation of NKA activity (d). *n* = 4~6. Scale bar, 30 *μ*m. Blue: DAPI. Green: NKA *α*1 staining. ^∗^*p* < 0.05 versus control group, †*p* < 0.05 versus Ang II alone group.

The immunofluorescence staining of *α*-actin was performed to reveal the H9c2 cardiomyocyte morphology (Figure 1(a)) and primary cultured neonatal mouse cardiomyocytes ([Supplementary-material supplementary-material-1]). It was found that Ang II (100 nM, 48 h) treatment significantly increased the cell size of cardiomyocytes, and this effect was attenuated by pretreatment with DR-Ab (2 *μ*M, 30 min) (Figure 1(b) and [Supplementary-material supplementary-material-1]). We also examined the mRNA expression of various hypertrophic biomarkers like atrial natriuretic peptide (ANP), brain natriuretic peptide (BNP), and beta-myosin heavy chain (*β*-MHC). Similar to what we observed in myocyte morphology, pretreatment with DR-Ab significantly attenuated Ang II-stimulated the above three hypertrophic biomarkers (Figure 1(c) and Figures [Supplementary-material supplementary-material-1]).

To study the underlying mechanisms, we first determined the effect of DR-Ab on NKA activity. As shown in Figure 1(d), DR-Ab attenuated Ang II-impaired NKA activity in the H9c2 cardiomyocytes (Figure 1(d)). We further examined the plasma membrane and total expression of NKA with Western blots and immunostaining. As shown in Figures 1(e)–1(g), treatment with Ang II reduced plasma membrane NKA expression (Figures 1(e) and 1(f) and [Supplementary-material supplementary-material-1]) and increased endosome NKA expression (Figures 1(g) and [Supplementary-material supplementary-material-1]), but had minor effect on its total protein expression. Pretreatment with DR-Ab reversed the effect of Ang II on plasma and endosome NKA expression. Taken together, our experiments indicated that DR-Ab inhibits plasma membrane NKA endocytosis. Our data imply that membrane NKA expression and activity are important in regulation of cell size when RAS is upregulated.

### 3.2. DR-Ab Alleviates Ang II-Induced Intracellular ROS Generation in H9c2 Cells

**Figure 2:**
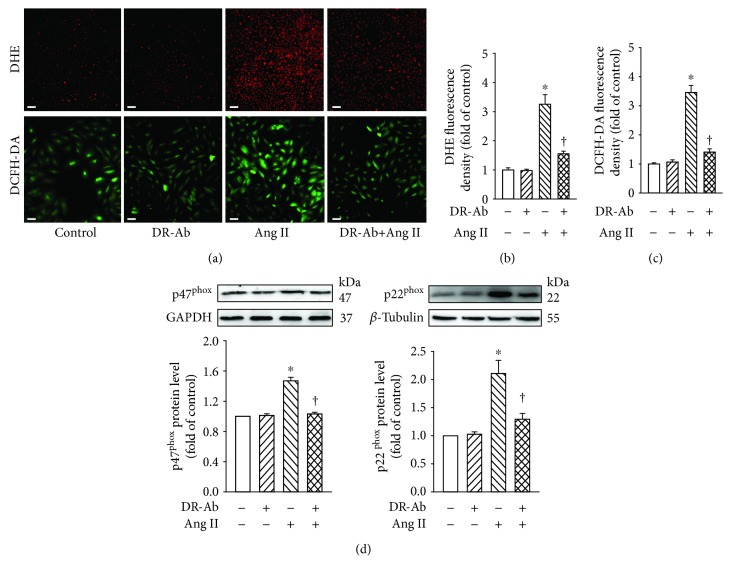
Effects of DR-Ab on Ang II-induced intracellular ROS generation in H9c2 cells. DR-Ab (2 *μ*M) was given 30 min before treatment with Ang II (100 nM) for 48 h. (a–c) Representative immunofluorescence image (a) and group data (b, c) showing that DR-Ab decreased ROS generation caused by Ang II. Red: DHE staining (a, upper). Scale bar, 100 *μ*m. Green: DCFH-DA staining (a, lower). Scale bar, 50 *μ*m. *n* = 6. (d) Effect of DR-Ab on the protein level of two subunits of NADPH oxidase: p22^phox^ and p47^phox^. *n* = 4‐6. ^∗^*p* < 0.05 versus control group, ^†^*p* < 0.05 versus Ang II alone group.

Oxidative stress plays an important role in Ang II-induced cardiomyopathy [2]. We first detected whether DR-Ab can affect Ang II-induced intracellular ROS production by using both DHE and DCFH-DA staining kits. As shown in Figures 2(a)–2(c), Ang II (100 nM, 48 h) significantly increased the generation of superoxide, hydroxyl, peroxyl, and other ROS. Pretreatment with DR-Ab (2 *μ*M, 30 min), which itself had no obvious effect, significantly reduced Ang II-induced intracellular ROS generation (Figures 2(a)–2(c)).

To examine the involvement of NADPH oxidase, we detected the protein expression of two subunits of NADPH oxidase (NOX2): p22^phox^ and p47^phox^. Western blotting analysis showed that treatment with Ang II upregulated the protein expression of these two proteins and this effect was attenuated by pretreatment with DR-Ab in both H9c2 and neonatal mouse cardiomyocytes (Figures 2(d) and [Supplementary-material supplementary-material-1]). Our data suggest that DR-Ab may inhibit NADPH oxidase activity in pathological situations.

### 3.3. DR-Ab Prevents Ang II-Induced Mitochondrial ROS and Energy Metabolic Dysfunction

**Figure 3:**
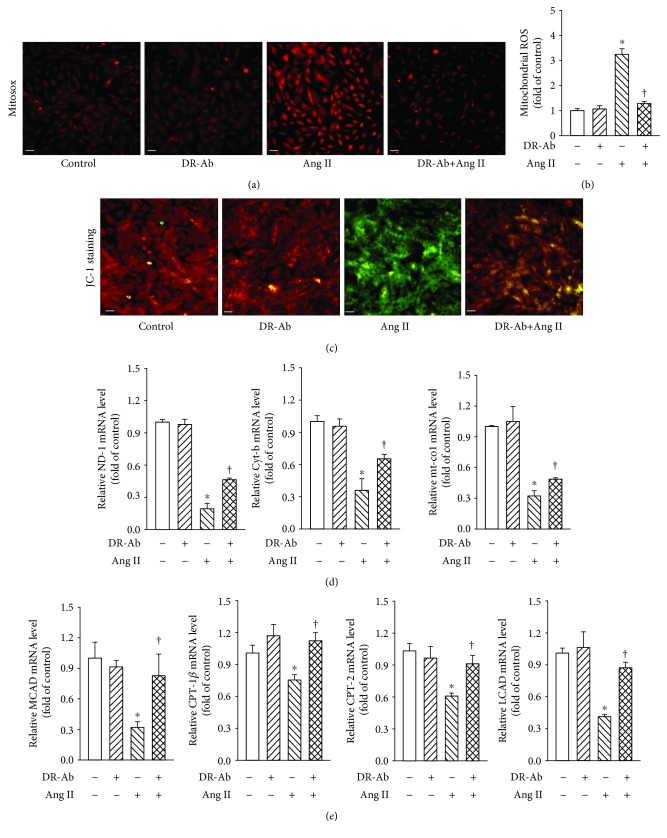
Effect of DR-Ab on Ang II-induced mitochondrial ROS (mit-ROS) generation and energy metabolic dysfunction. (a, b) Representative image (a) and group data (b) showing that DR-Ab decreased Ang II-induced mit-ROS generation. Red: Mit-ROS. Scale bar, 50 *μ*m. *n* = 4‐6. (c) Representative JC-1 staining showing that DR-Ab reversed mitochondrial membrane potential loss caused by Ang II. Red: aggregate. Green: monomer. Scale bar, 50 *μ*m. (d) qRT-PCR analysis showing that DR-Ab increased the mRNA expression of mitochondrial encoded genes (ND-1, Cyt-b, and mt-co1) in Ang II-treated cells. *n* = 4. (e) qRT-PCR analysis showing the effect of DR-Ab on the mRNA expression of fatty acid oxidation related genes (CPT-1*β*, CPT-2, LCAD, and MCAD). *n* = 4. ^∗^*p* < 0.05 versus control group, ^†^*p* < 0.05 versus Ang II alone group.

We further studied mitochondrial ROS generation with MitoSOX™ Red staining. As shown in Figures 3(a) and 3(b), Ang II significantly increased mitochondrial ROS generation in the mitochondria, and this effect was reversed by pretreatment with DR-Ab. The mitochondrial permeability transition, an important step in the induction of cellular apoptosis, was also determined using the unique fluorescent cationic dye, JC-1. It was found that Ang II induced loss of red JC-1aggregate fluorescence, and only green monomer fluorescence was detected in the cytoplasm of these cells (Figure 3(c)). This effect was also reversed by DR-Ab treatment.

We continued to study the mRNA levels of mitochondrial DNA-encoded genes including ND-1, cyt-b, and mt-co1. Real-time PCR analysis showed that pretreatment with DR-Ab significantly attenuated Ang II-suppressed expression of these genes (Figure 3(d)). Our data imply that DR-Ab recovered impaired mitochondrial function induced by Ang-II.

Fatty acid oxidation (FAO) is one of the pivotal mechanisms involved in the development of cardiomyopathy [25]. We also studied whether DR-Ab can affect fatty acid metabolism in Ang-II-induced H9C2 cardiomyocyte damage. As shown in Figure 3(e), Ang II-significantly reduced the mRNA expression of FAO-related genes including CPT-1*β*, CPT-2, long-chain acyl-CoA dehydrogenase (LCAD), and medium-chain acyl-CoA dehydrogenase (MCAD), and these effects were reversed by pretreatment with DR-Ab. These data suggest that DR-Ab may improve Ang II-induced impaired fatty acid oxidation.

### 3.4. DR-Ab Protects H9c2 Cardiomyocytes against Ang II-Induced Hypertrophy via Activation of AMPK/Sirt-3/PPAR*γ* Signaling Pathway

**Figure 4:**
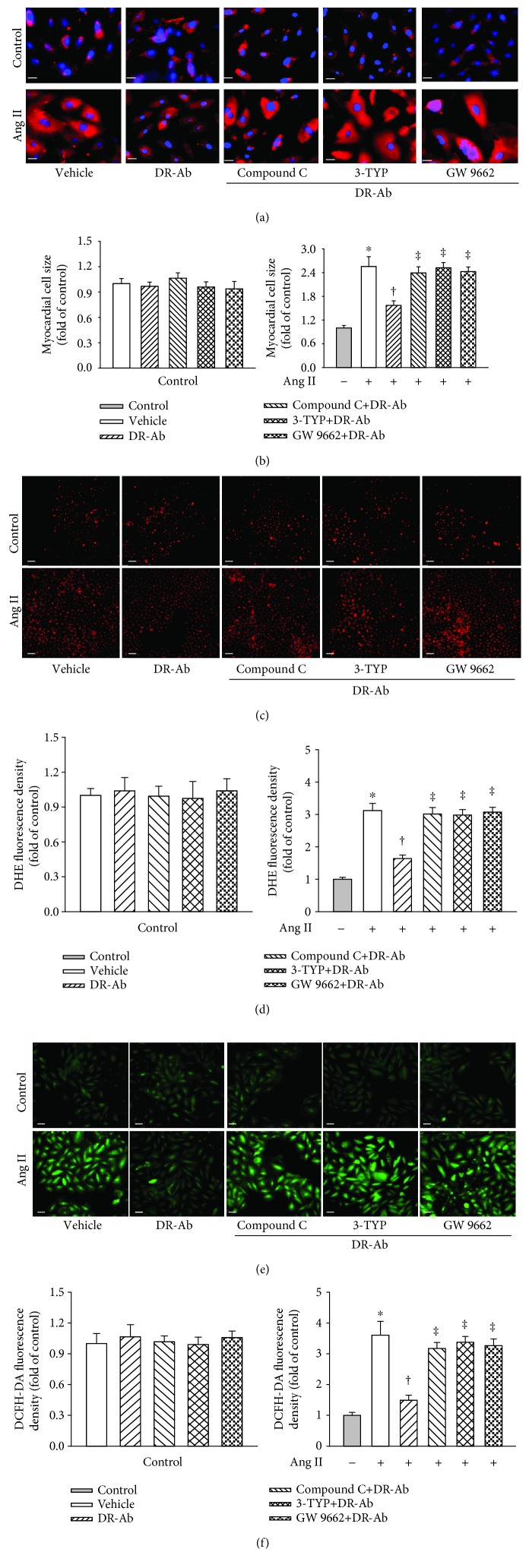
Effect of DR-Ab on myocyte hypertrophy and intracellular ROS generation in Ang II-treated H9c2 in the presence and absence of compound C (40 *μ*M, a selective AMPK inhibitor), 3-TYP (50 *μ*M, a selective Sirt3 inhibitor), or GW9662 (10 *μ*M, a PPAR*γ* antagonist). Cells were treated with these inhibitors for 30 min before DR-Ab (2 *μ*M, 30 min) and Ang II (100 nM, 48 h). (a, b) Representative immunofluorescence staining (a) and group data (b) showing that blockade of AMPK, Sirt3, or PPAR*γ* abolished the effect of DR-Ab on cell size. Red: *α*-actinin. Blue: DAPI. Scale bar, 25 *μ*m. *n* = 4‐6. (c–f) Representative image (c, e) and group data (d, f) showing that blockade of AMPK, Sirt3, or PPAR*γ* promoted the intracellular ROS which were decreased by DR-Ab in Ang II-induced cells. (c) Red: DHE relative fluorescence density. Scale bar, 100 *μ*m. (e) Green: DCFH-DA staining. Scale bar, 50 *μ*m. *n* = 4‐6. ^∗^*p* < 0.05 versus control, ^†^*p* < 0.05 versus Ang II alone group, ^‡^*p* < 0.05 versus Ang II+DR-Ab group.

**Figure 5:**
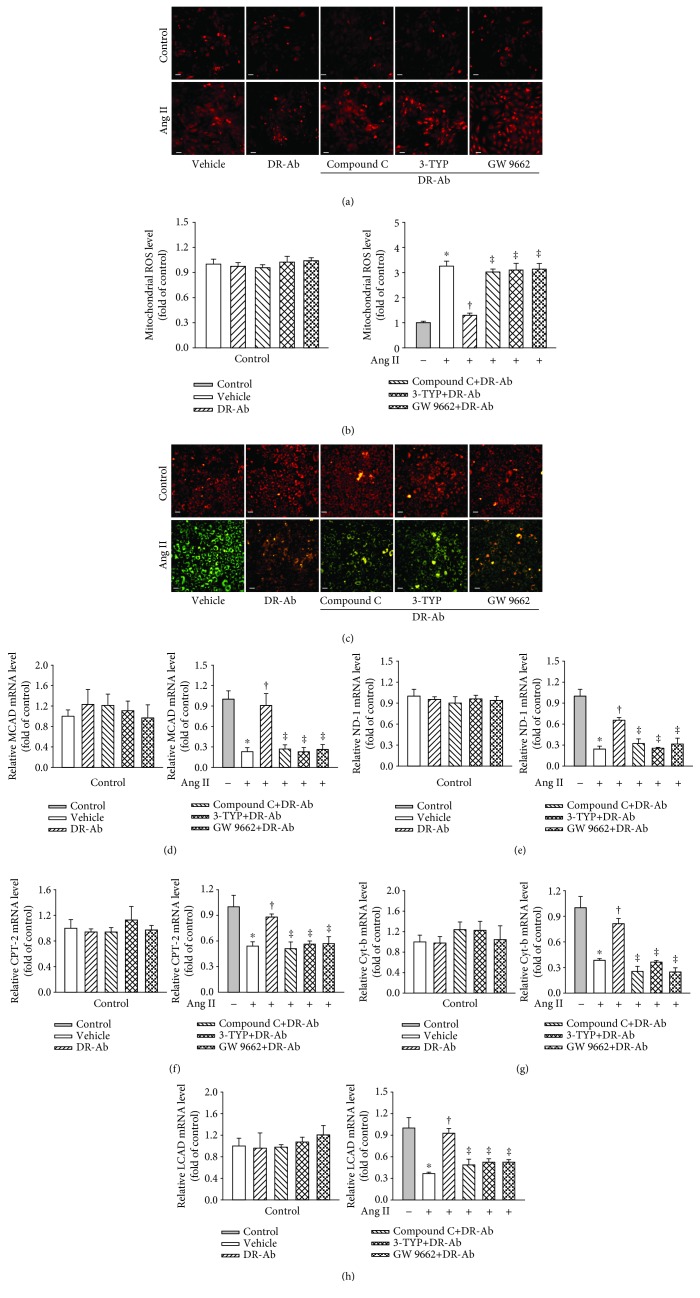
Effects of DR-Ab on mit-ROS production and energy metabolic dysfunction in Ang II-treated cells in the presence and absence of inhibitors of AMPK, Sirt3, or PPAR*γ*. (a, b) Representative image (a) and statistic data (b) showing that blockade of AMPK, Sirt3, or PPAR*γ* with their inhibitors abolished the protective effect of DR-Ab on mit-ROS production. Red: mit-ROS. Scale bar, 50 *μ*m. *n* = 4‐6. (c) JC-1 staining showing that blockade of AMPK, Sirt3, or PPAR*γ* reversed the effect of DR-Ab on mitochondrial membrane potential. Red: aggregate. Green: monomer. Scale bar, 50 *μ*m. (d–h) qRT-PCR analysis showing that blockade of AMPK, Sirt3, or PPAR*γ* abolished the effects of DR-Ab on the mRNA expression of ND-1, Cyt-b, CPT-2, LCAD, and MCAD. *n* = 4‐6. ^∗^*p* < 0.05 versus control, ^†^*p* < 0.05 versus Ang II alone group, ^‡^*p* < 0.05 versus Ang II+DR-Ab group.

It is well known that the AMPK/Sirt-3/PPAR*γ* signaling pathway participates in Ang II-induced cardiomyocyte hypertrophy [26–34]. In this study, we also tested the involvement of this pathway in the effect of DR-Ab. We first repeated the effects of DR-Ab on cell morphology (Figures 4(a) and 4(b)), intracellular (Figures 4(c)–4(f)) and mitochondrial ROS (Figures 5(a) and 5(b)) generation, mitochondrial membrane potential loss (Figure 5(c)), and mitochondrial function-related gene level (Figures 5(d)–5(h)) in the presence and absence of compound C, an AMPK inhibitor, 3-TYP, a selective Sirt3 inhibitor, and GW9662, a PPAR*γ* antagonist. As shown in Figures 4 and 5, all these inhibitors abolished the protective effects of DR-Ab. Our data suggest that the AMPK/Sirt-3/PPAR*γ* signaling pathway mediates the cardioprotective effects of DR-Ab.

**Figure 6:**
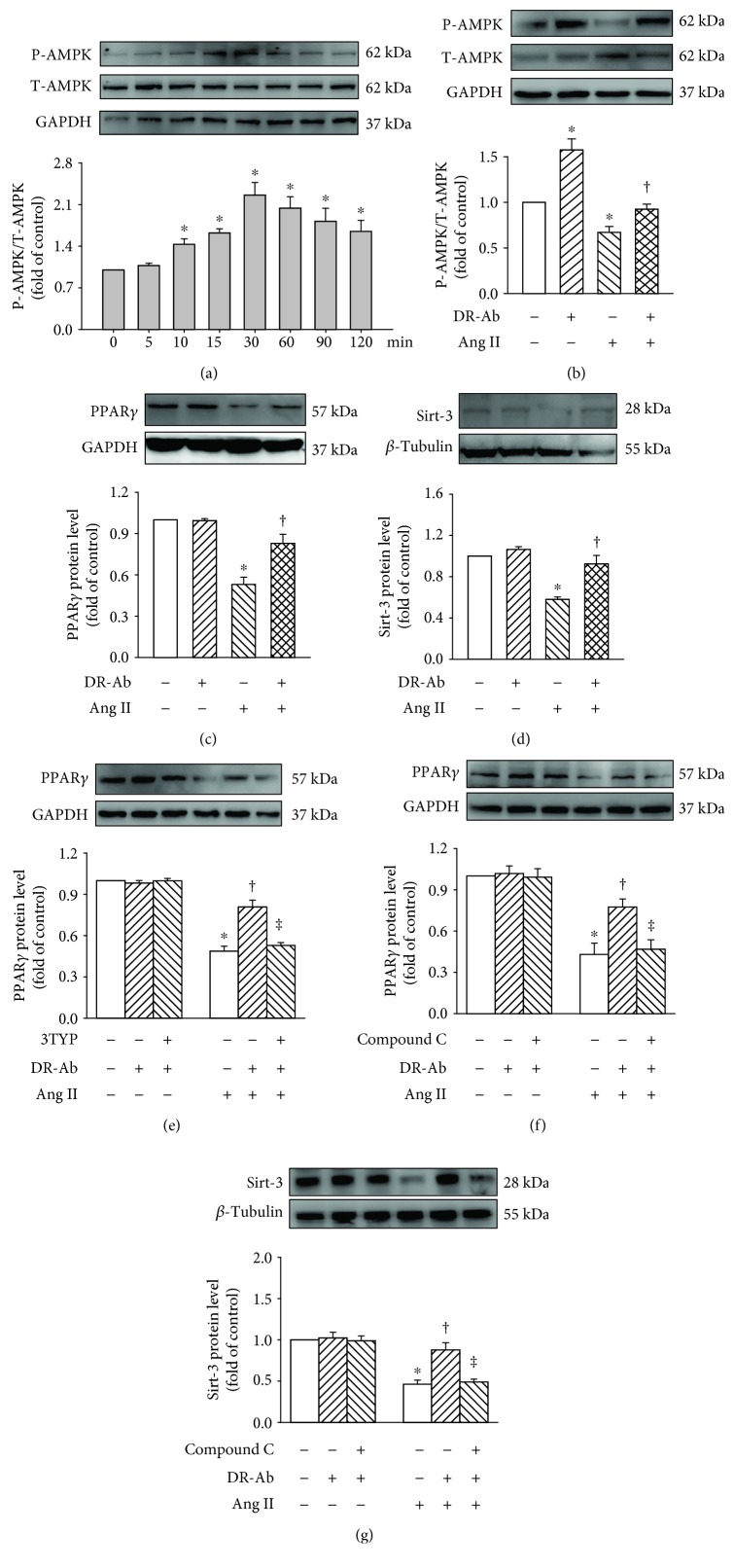
Western blotting analysis showing that DR-Ab stimulated AMPK/Sirt-3/PPAR*γ* signaling pathway. (a) Time-course study showing the effect of DR-Ab on AMPK phosphorylation. (b–d) DR-Ab reversed the effect of Ang II on p-AMPK (b), PPAR*γ* (c), & Sirt-3 (d). *n* = 4‐6. (e) 3-TYP eliminated DR-Ab effect on the PPAR*γ* level in Ang II-treated cells. *n* = 4. (f, g) Compound C abolished the effect of DR-Ab on the protein expression of PPAR*γ* (f) and Sirt-3 (g) in Ang II-treated cells. *n* = 4‐6. ^∗^*p* < 0.05 versus control group, ^†^*p* < 0.05 versus Ang II alone group, ^‡^*p* < 0.05 versus Ang II+ DR-Ab.

To further confirm the involvement of this signaling pathway, we observed the effect of DR-Ab on AMPK phosphorylation (P-AMPK). A time-course study showed that DR-Ab obviously increased P-AMPK level and the strongest effect was observed when cells were treated with DR-Ab for 30 min (Figure 6(a)). For this reason, DR-Ab reversed Ang II-suppressed P-AMPK (Figure 6(b)). To study the signaling cascade, compound C, an AMPK inhibitor, was used. As shown in Figures 6(f) and 6(g), compound C abolished the effect of DR-Ab on both Sirt-3 and PPAR*γ*. Moreover, Ang II treatment significantly reduced the protein levels of PPAR*γ* and Sirt-3 (Figures 6(c) and 6(d)). These effects were significantly attenuated by incubation with DR-Ab. Interestingly, treatment with 3-TYP, a selective Sirt3 inhibitor, reversed the effect of DR-Ab on PPAR*γ* protein expression (Figure 6(e)). Taken together, DR-Ab protects H9c2 cardiomyocytes against Ang II-induced hypertrophy may via activate the AMPK/Sirt-3/PPAR*γ* signaling pathway.

## 4. Discussion

Ang II, a key component of renin-angiotensin system (RAS), is crucial in cardiovascular physiology and pathology [35]. The increased circulating Ang II level and activated RAS are closely associated with cardiovascular diseases such as cardiac hypertrophy [36] and heart failure [37]. Therefore, Ang II is widely used to mimic the pathology of clinical cardiac hypertrophy. An important function of NKA is to regulate cell volume [38, 39]. Recently, NKA expression and activity were also found to be closely regulated by Ang II [14, 40–42]. For instance, Ang II can inhibit NKA activity via induction of NADPH oxidase-derived O_2_^∗^^−15^. Molkentin's group also reported that overexpression of NKA successfully protects the heart against pathological cardiac hypertrophy and remodeling [43]. We previously reported that DR-Ab protects the heart against oxidative and ischemic injury [10, 12]. In this study, we demonstrated for the first time that DR-Ab prevented Ang II-induced myocyte hypertrophy through observing myocyte size, cell morphology, ROS generation, and mitochondrial functions.

We first investigated whether Ang II can regulate NKA expression and function in this study. It was found that Ang II treatment significantly reduced both plasma expression and activity of NKA. To study whether Ang II-induced pathology is caused by impairment of NKA, we pretreated the cells with DR-Ab which stimulates NKA activity. As expected, DR-Ab improved cardiomyocytes hypertrophy induced by Ang II. The enlarged cell size induced by Ang II was recovered to nearly normal cell after treatment with DR-Ab. Our results suggest that DR-Ab protect cells against Ang II-induced cells injury through preservation of membrane NKA activity, which helps to maintain resting potential, ion transport, and regulates cellular volume.

Oxidative stress plays an important role in Ang II-induced cardiomyopathy [44]. Recent studies have revealed that NKA is one of the target proteins of ROS [45, 46]. Moreover, it was found that NADPH oxidase is crucial for the inhibited NKA activity in cardiac myocytes treated with Ang II [13]. For the above reasons, we first examined whether DR-Ab can protect cardiomyocytes through inhibition of NADPH oxidase. We found that DR-Ab markedly attenuated Ang II-induced intracellular ROS generation through inhibition of NADPH oxidase. This effect was achieved by inhibition of the upregulated protein expression of p22^phox^ and p47^phox^ caused by Ang II.

Mitochondrial dysfunction also produces high levels of ROS. Multiple experiments were therefore performed to test the mitochondrial functions. We found that Ang II treatment largely increased mitochondrial ROS production and decreased mitochondrial membrane potential. This is consistent with previous studies [47]. We further studied its effects on mitochondrial DNAs, which encode proteins for the electron transport chain and then produce the majority of cellular energy [48, 49]. Our results showed that Ang II notably decreased the mRNA levels of ND-1, cyt-b, and mt-co1. Metabolic derangement is a signature in pathological cardiac hypertrophy [50]. Ang II also significantly inhibited the mRNA levels of CPT-1*β*, CPT-2, LCAD, and MCAD, all of which are important in mitochondrial oxidative phosphorylation and fatty acid metabolism. Interestingly, all the above effects caused by Ang II were significantly reversed by the pretreatment with DR-Ab.

DR-Ab is an antibody targeted at the 4^th^ extracellular domain of *α*-subunit of NKA. It remains unclear why and how DR-Ab protects mitochondrial functions by binding to NKA. As NKA also serve as a signaling protein [28], we studied the signaling mechanisms underlying the protective effect of DR-Ab. Previous studies revealed that Ang II-induced cardiac hypertrophy is mediated by AMPK- [29–31, 51, 52], Sirt3- [32–34], and PPAR*γ*- [26–28] dependent mechanisms. Therefore, there is a close relationship between NKA and AMPK [53–56]. On the one hand, activation of AMPK has been reported to regulate the activity and cell surface abundance of NKA [57]. On the other hand, ouabain blocks the carbachol-induced phosphorylation and activation of AMPK [58]; thus, activation of NKA also stimulates AMPK activity [59]. For this reason, we studied the effect of DR-Ab on AMPK activity and found that DR-Ab promoted AMPK phosphorylation. Activation of AMPK has been shown to stimulate Sirt-3 [60–62], and then activated Sirt-3 affects PPAR*γ* [32] which has been proved in participating in Ang II-induced myocyte hypertrophy [26–28]. In our study, we confirmed the involvement of the Sirt-3/PPAR*γ* pathway with pharmacological manipulation. Western botting analysis also confirmed that activation of Sirt-3/PPAR*γ* is secondary to that of AMPK. Our data suggest that the AMPK/Sirt-3/PPAR*γ* signaling pathway mediates the protective effects of DR-Ab against Ang II-induced H9c2 cardiomyocyte damage.

**Figure 7:**
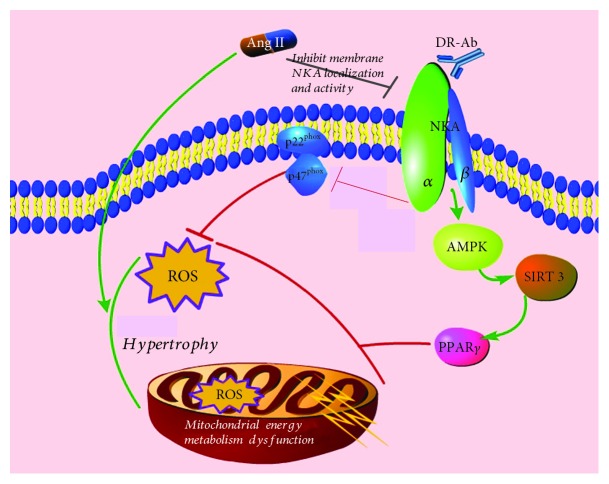
Schematic illustration showing the mechanisms for the protective effects of DR-Ab. DR-Ab protects against Ang II-induced cell injury by stabilization of membrane NKA and stimulation of its activity. This helps to maintain the normal intracellular ion homeostasis, thus reserves the cell size. DR-Ab inhibits NADPH oxidase activity by downregulation of p22^phox^ and p47^phox^ expression. Meanwhile, DR-Ab inhibits mitochondrial ROS generation and preserves mitochondrial function through stimulation of the AMPK/Sirt-3/PPAR*γ* signaling pathway.

In summary, as shown in Figure 7, we found for the first time that DR-Ab prevents Ang II-induced H9c2 cardiomyocyte hypertrophy. This protective effect is mediated by activation of the AMPK/Sirt-3/PPAR*γ* signaling pathway and stabilization of membrane NKA expression. Our results suggest a novel mechanism and therapeutic strategy in the treatment of cardiac hypertrophy and associated oxidative injury.

## Figures and Tables

**Table 1:** 

Gene (rat)	Primer sequences (5′-3′)
GAPDH	Forward: AGGAGTAAGAAACCCTGGAC
	Reverse: CTGGGATGGAATTGTGAG
ANF	Forward: CCGTATACAGTGCGGTGTCC
	Reverse: CAGAGAGGGAGCTAAGTGCC
BNP	Forward: AGCTGCTTTGGGCAGAAGAT
	Reverse: AAAACAACCTCAGCCCGTCA
*β*-MHC	Forward: GACAACGCCTATCAGTACATG
	Reverse: TGGCAGCAATAACAGCAAAA
ND1	Forward: AAGCGGCTCCTTCTCCCTACAAAT
	Reverse: GAAGGGAGCTCGATTTGTTTCTGC
Cytb	Forward: GCAGCTTAACATTCCGCCCAATCA
	Reverse: TGTTCTACTGGTTGGCCTCCGATT
mt-co1	Forward: AAGGTTTGGTCCTGGCCTTA
	Reverse: GGCAAGGCGTCTTGAGCTAT
CPT-1*β*	Forward: TCAAGGTTTGGCTCTATGAGGGCT
	Reverse: TCCAGGGACATCTTGTTCTTGCCA
CPT-2	Forward: TCCTGCATACCAGCAGATGAACCA
	Reverse: TATGCAATGCCAAAGCCATCAGGG
LCAD	Forward: AATGGGAGAAAGCCGGAGAAGTGA
	Reverse: GATGCCGCCATGTTTCTCTGCAAT
MCAD	Forward: CTGCTCGCAGAAATGGCGATGAAA
	Reverse: CAAAGGCCTTCGCAATAGAGGCAA

Gene (mouse)	Primer sequences (5′-3′)
*β*-Actin	Forward: CCGTGAAAAGATGACCCAGA
	Reverse: CTGGGATGGAATTGTGAG
ANP	Forward: ACCTGCTAGACCACCTGGAG
	Reverse: CCTTGGCTGTTATCTTCGGTACCGG
BNP	Forward: GAGGTCACTCCTATCCTCTGG
	Reverse: GCCATTTCCTCCGACTTTTCTC
*β*-MHC	Forward: CCGAGTCCCAGGTCAACAA
	Reverse: CTTCACGGGCACCCTTGGA

## Data Availability

The derived data used to support the findings of this study are included within the article. The raw data used to support the findings of this study are available from the corresponding author upon request.

## Supplementary Materials

Supplementary Materials Figure S1: Effects of DR-Ab on Ang II-induced hypertrophy in the neonatal mouse cardiomyocytes. DR-Ab (2 *μ*M) was given 30 min before treatment with Ang II (100 nM) for 48 h. (A-B) Representative immunofluorescence staining (A) and group data (B) showing that DR-Ab reversed enlarged cell size caused by Ang II. Green: NKA *α*1. Blue: DAPI. Scale bar, 30 *μ*m. *n* = 6. (C-E) qRT-PCR analysis showing the mRNA levels of ANP, BNP, and *β*-MHC. *n* = 4. (F-G) DR-Ab reversed Ang II-induced loss of plasma membrane NKA *α*1 (A&F) and increase of endosome NKA *α*1 (G). *n* = 4‐6. (H-I) Effect of DR-Ab on the protein level of two subunits of NADPH oxidase: p22^phox^ and p47^phox^. *n* = 4‐6. ^∗^*p* < 0.05 versus control group, ^†^*p* < 0.05 versus Ang II alone group.
