# Supplementary material for: Stimulation of Na+/K+-ATPase with an Antibody against Its 4th Extracellular Region Attenuates Angiotensin II-Induced H9c2 Cardiomyocyte Hypertrophy via an AMPK/SIRT3/PPARγ Signaling Pathway
Source: Oxid Med Cell Longev. 2019 Sep 15;2019:4616034. doi: 10.1155/2019/4616034 (PMC6766118; doi:10.1155/2019/4616034)
Supplement: Supplementary Materials — Figure S1: Effects of DR-Ab on Ang II-induced hypertrophy in the neonatal mouse cardiomyocytes. DR-Ab (2 μM) was given 30 min before treatment with Ang II (100 nM) for 48 h. (A-B) Representative immunofluorescence staining (A) and group data (B) showing that DR-Ab reversed enlarged cell size caused by Ang II. Green: NKA α1. Blue: DAPI. Scale bar, 30 μm. n = 6. (C-E) qRT-PCR analysis showing the mRNA levels of ANP, BNP, and β-MHC. n = 4. (F-G) DR-Ab reversed Ang II-induced loss of plasma membrane NKA α1 (A&F) and increase of endosome NKA α1 (G). n = 4‐6. (H-I) Effect of DR-Ab on the protein level of two subunits of NADPH oxidase: p22phox and p47phox. n = 4‐6. ∗p < 0.05 versus control group, †p < 0.05 versus Ang II alone group. [file 4616034.f1.docx]

**Supplementary material**

**
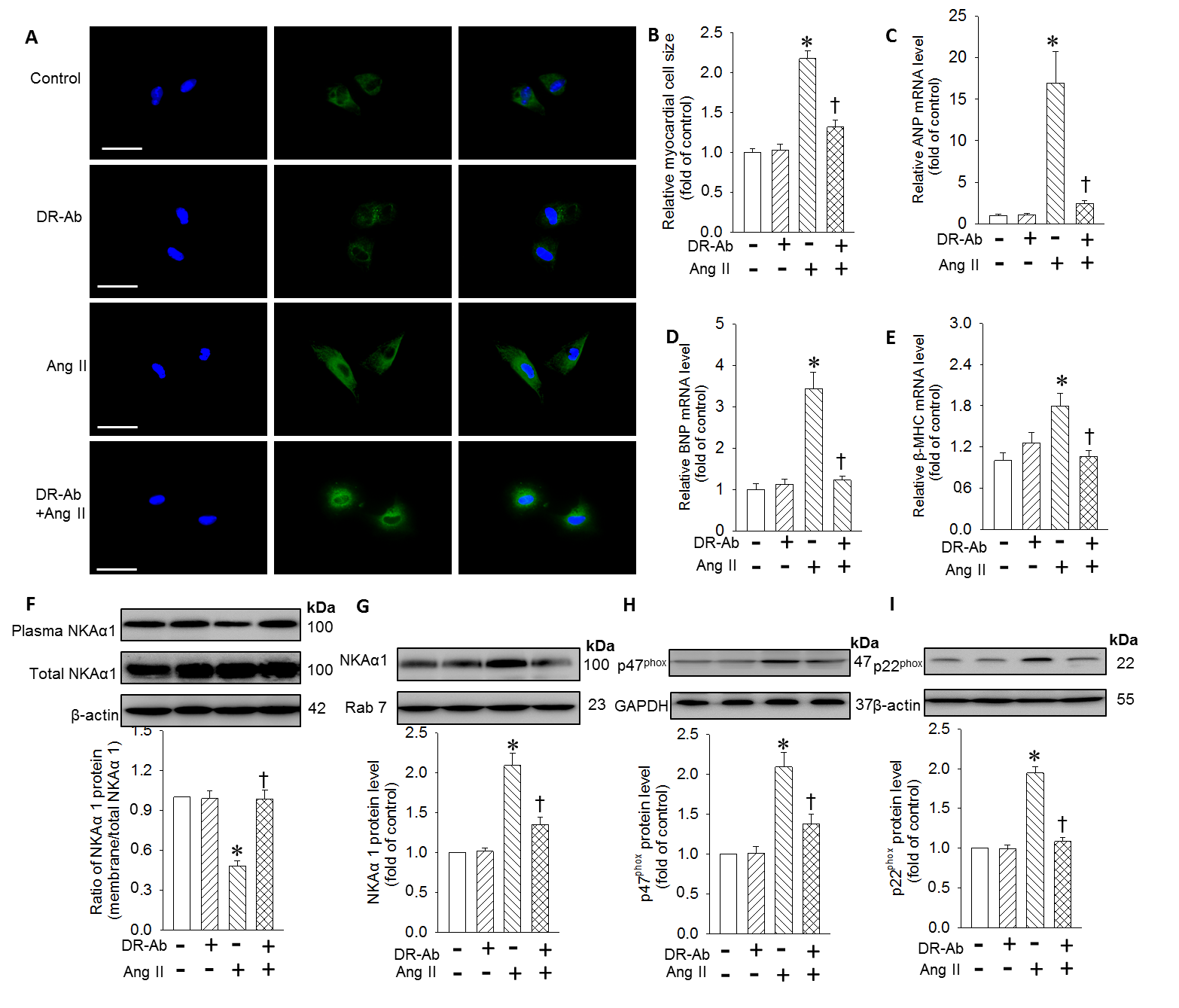
**

**Figure S1. Effects of DR-Ab on Ang II-induced hypertrophy in the neonatal mouse cardiomyocytes.** DR-Ab (2μM) was given 30 min before treatment with Ang Ⅱ (100 nM) for 48 h. (A-B) Representative immunofluorescence staining (A) and group data (B) showing that DR-Ab reversed enlarged cell size caused by Ang II. Green: NKA α1. Blue: DAPI. Scale bar, 30μm. n=6. (C-E) qRT-PCR analysis showing the mRNA levels of ANP, BNP and β-MHC. n=4 (F-G) DR-Ab reversed Ang II-induced loss of plasma membrane NKA α1 (A&F) and increase of endosome NKA α1(G) ,n=4-6. (H-I) Effect of DR-Ab on the protein level of two subunits of NADPH oxidase: p22^phox^ and p47^phox^. n= 4-6. *p<0.05 versus Control group, † p<0.05 versus Ang Ⅱ alone group.
